# Correction: Contextual validation of HEMLEM tool used for measuring clinical micro-learning environments

**DOI:** 10.1371/journal.pone.0340415

**Published:** 2026-01-05

**Authors:** Khizar Ansar Malik, Ayesha Fahim, Abeer Anjum, Saria Khalid, Shafaq Malik, Ahsan Sethi

The fourth author’s name is spelled incorrectly. The correct name is: Saria Khalid. The correct citation is: Malik KA, Fahim A, Anjum A, Khalid S, Malik S, Sethi A (2025) Contextual validation of HEMLEM tool used for measuring clinical micro-learning environments. PLoS One 20(12): e0337641. https://doi.org/10.1371/journal.pone.0337641
