# Supplementary material for: Contextual validation of HEMLEM tool used for measuring clinical micro-learning environments
Source: PLoS One. 2025 Dec 10;20(12):e0337641. doi: 10.1371/journal.pone.0337641 (PMC12694844; doi:10.1371/journal.pone.0337641)
Supplement: S1 Table — (DOCX) [file pone.0337641.s001.docx]

# SUPPLEMENTARY FILE 1

# DESCRIPTIVE STATISTICS AND DEMOGRAPHICS SUMMARY

## Table 1. Participant Demographics

Gender Distribution

| Gender | Count | Percentage |
| --- | --- | --- |
| Female | 349.0 | 55.6% |
| Male | 279.0 | 44.4% |

Discipline Distribution

| Discipline | Count | Percentage |
| --- | --- | --- |
| MBBS | 402.0 | 64.0% |
| BDS | 226.0 | 36.0% |

Year of Study Distribution

| Year | Count | Percentage |
| --- | --- | --- |
| MBBS 3^rd^ year | 125.0 | 19.9% |
| MBBS 4^th^ year | 139.0 | 22.1% |
| MBBS final year | 138.0 | 22.0% |
| BDS 3^rd^ year | 118.0 | 18.8% |
| BDS 4^th^ year | 108.0 | 17.2% |

## Table 2. Descriptive Statistics for HEMLEM 2.0 Items

| Item | Mean | SD | Min | Max | Skew | Kurtosis |
| --- | --- | --- | --- | --- | --- | --- |
| This placement had a welcoming, friendly, and open atmospher | 2.28 | 0.97 | 1.0 | 5.0 | 0.75 | 0.52 |
| There was a culture where I felt free to ask questions or ma | 2.41 | 1.02 | 1.0 | 5.0 | 0.84 | 0.26 |
| Facilitators on this placement were enthusiastic about teach | 2.5 | 1.0 | 1.0 | 5.0 | 0.59 | 0.06 |
| My facilitator showed an interest in me. | 2.72 | 1.09 | 1.0 | 5.0 | 0.29 | -0.49 |
| My input was valued on this placement. | 2.67 | 1.01 | 1.0 | 5.0 | 0.56 | -0.02 |
| I was provided with regular, useful, and supportive feedback | 2.7 | 1.04 | 1.0 | 5.0 | 0.42 | -0.45 |
| I had opportunity to apply my previous knowledge in this pla | 2.34 | 0.9 | 1.0 | 5.0 | 0.73 | 0.57 |
| My knowledge and skills were developed on this placement. | 2.49 | 1.0 | 1.0 | 5.0 | 0.59 | 0.0 |
| This placement helped me put theory into practice. | 2.79 | 1.13 | 1.0 | 5.0 | 0.35 | -0.78 |
| I was able to meet my learning objectives on this placement. | 2.51 | 1.02 | 1.0 | 5.0 | 0.66 | -0.0 |
| I had the opportunity to understand how to deal with the pat | 2.75 | 1.2 | 1.0 | 5.0 | 0.46 | -0.75 |
| I was given tasks suitable for my stage of training on this | 2.62 | 1.07 | 1.0 | 5.0 | 0.59 | -0.23 |
