# Supplementary material for: Contextual validation of HEMLEM tool used for measuring clinical micro-learning environments
Source: PLoS One. 2025 Dec 10;20(12):e0337641. doi: 10.1371/journal.pone.0337641 (PMC12694844; doi:10.1371/journal.pone.0337641)
Supplement: S2 Table — (DOCX) [file pone.0337641.s002.docx]

**SUPPLEMENTARY FILE 2:**

**Professional Profile of the experts in content validation**

| **Sr. No.** | **Gender** | **Professional Field** | **Specialty** | **Years of Professional Experience** | **Academic Level** |
| --- | --- | --- | --- | --- | --- |
|  | Female | Medicine | Gynaecology, Associate Professor | 17 | MBBS, FCPS |
|  | Male | Medicine | General Medicine, University Professor | 22 | MBBS, MME, FCPS |
|  | Male | Medicine | General Surgery, Assistant Professor | 10 | MBBS, CHPE, FCPS |
|  | Female | Medicine | Paediatrics, Associate Professor | 12 | MBBS, FCPS |
|  | Male | Medicine | General Medicine, University Professor | 19 | MBBS, MHPE, FCPS |
|  | Female | Medicine | Obstetrician-Gynaecologist. Consultant | 16 | MBBS, FCPS |
|  | Male | Medicine | Anaesthetist | 15 | MBBS, FCPS |
|  | Female | Medicine | Dermatologist | 11 | MBBS, FCPS |
|  | Male | Medicine | Paediatric Surgeon, Consultant | 15 | MBBS, FCPS |
|  | Male | Medicine | Cardiologist | 18 | MBBS, FCPS |
|  | Male | Dentistry | Orthodontist, University Professor | 20 | BDS, MHPE, FCPS |
|  | Male | Dentistry | Prosthodontist, Associate Professor | 10 | BDS, MDS |
|  | Female | Dentistry | Endodontist, Associate Professor | 13 | BDS, MHPE, FCPS |
|  | Male | Dentistry | Periodontist, Associate Professor | 11 | BDS, CHPE, MSc |
|  | Male | Dentistry | Periodontist, Assistant Professor | 10 | BDS, FCPS |
|  | Male | Dentistry | Oral Surgeon, University Professor | 22 | BDS, MDS, MSc |
|  | Female | Dentistry | General Dentist | 10 | BDS |
|  | Female | Dentistry | Orthodontist | 15 | BDS, FCPS |
|  | Male | Dentistry | Oral Surgeon, Consultant | 18 | BDS, FCPS |
|  | Female | Dentistry | General Dentist | 10 | BDS |
