# Supplementary material for: Contextual validation of HEMLEM tool used for measuring clinical micro-learning environments
Source: PLoS One. 2025 Dec 10;20(12):e0337641. doi: 10.1371/journal.pone.0337641 (PMC12694844; doi:10.1371/journal.pone.0337641)
Supplement: S3 Table — (DOCX) [file pone.0337641.s003.docx]

**SUPPLEMENTARY FILE 3:**

**Revised version of HEMLEM 2.0**

| 1. This department had a welcoming, friendly, and open atmosphere. |
| --- |
| 1. There was a culture where I felt free to ask questions or make comments on this department. |
| 1. Staff in this department were enthusiastic about teaching. |
| 1. My supervisor showed an interest in me. |
| 1. My input was valued in this department. |
| 1. I was provided with regular, useful, and supportive feedback during my rotation in this department. |
| 1. I had the opportunity to apply my previous knowledge in this department. |
| 1. My knowledge and skills were developed in this department. |
| 1. This department helped me put theory into practice. |
| 1. I was able to meet my learning objectives in this department. |
| 1. I had the opportunity to deal with the patient as a whole in this department. |
| 1. I was given tasks suitable for my stage of training in this department. |
