# Supplementary material for: Contextual validation of HEMLEM tool used for measuring clinical micro-learning environments
Source: PLoS One. 2025 Dec 10;20(12):e0337641. doi: 10.1371/journal.pone.0337641 (PMC12694844; doi:10.1371/journal.pone.0337641)
Supplement: S6 Table — (DOCX) [file pone.0337641.s006.docx]

**SUPPLEMENTARY FILE 6:**

**CONFIRMATORY FACTOR ANALYSIS AND MODEL FIT COMPARISON**

**Model Specification**

**3-Factor Model (Theory-driven):**

**Supervision**: Items Q1-Q4

**Autonomy**: Items Q5-Q8

**Atmosphere**: Items Q9-Q12

**Table S6.1: Descriptive Statistics**

| **(N =**  **628) Item** | **Mean** | **SD** | **Skewness** | **Kurtosis** | **Min** | **Max** |
| --- | --- | --- | --- | --- | --- | --- |
| Q1 | 3.308 | 1.035 | -0.430 | -0.454 | 1 | 5 |
| Q2 | 3.279 | 1.097 | -0.287 | -0.521 | 1 | 5 |
| Q3 | 3.487 | 1.009 | -0.572 | 0.008 | 1 | 5 |
| Q4 | 3.341 | 1.019 | -0.570 | -0.043 | 1 | 5 |
| Q5 | 3.666 | 0.906 | -0.759 | 0.585 | 1 | 5 |
| Q6 | 3.523 | 0.986 | -0.622 | 0.101 | 1 | 5 |
| Q7 | 3.234 | 1.125 | -0.371 | -0.742 | 1 | 5 |
| Q8 | 3.510 | 1.009 | -0.672 | 0.063 | 1 | 5 |
| Q9 | 3.737 | 0.967 | -0.791 | 0.577 | 1 | 5 |
| Q10 | 3.591 | 1.021 | -0.841 | 0.224 | 1 | 5 |
| Q11 | 3.269 | 1.197 | -0.460 | -0.755 | 1 | 5 |
| Q12 | 3.390 | 1.066 | -0.597 | -0.258 | 1 | 5 |

**Table S6.2: Model Fit Indices**

| **Model** | **χ²** | **df** | **χ²/df** | **CFI** | **TLI** | **RMSEA [90% CI]** | **SRMR** |
| --- | --- | --- | --- | --- | --- | --- | --- |
| One-factor | 312.45 | 54 | 5.79 | 0.802 | 0.778 | 0.095 [0.085-0.106] | 0.087 |
| Three-factor | 142.36 | 51 | 2.79 | 0.932 | 0.914 | 0.081 [0.065-0.097] | 0.058 |
| Bifactor | 126.78 | 42 | 3.02 | 0.943 | 0.918 | 0.077 [0.060-0.094] | 0.052 |

**Table S6.3: Factor Correlations**

|  | **Supervision** | **Autonomy** | **Atmosphere** |
| --- | --- | --- | --- |
| Supervision | 1.000 |  |  |
| Autonomy | 0.721 | 1.000 |  |
| Atmosphere | 0.684 | 0.692 | 1.000 |

**Table S6.4: Reliability Analysis**

| **Scale/Subscale** | **Items** | **Cronbach's α** | **95% CI** |
| --- | --- | --- | --- |
| Total Scale | Q1-Q12 | 0.897 | [0.883-0.910] |
| Supervision | Q1-Q4 | 0.787 | [0.758-0.814] |
| Autonomy | Q5-Q8 | 0.780 | [0.750-0.807] |
| Atmosphere | Q9-Q12 | 0.736 | [0.702-0.767] |

**Group-Specific Reliability**

**By Gender**

| **Group** | **N** | **Total α** | **Supervision α** | **Autonomy α** | **Atmosphere α** |
| --- | --- | --- | --- | --- | --- |
| Male | 279 | 0.891 | 0.758 | 0.791 | 0.745 |
| Female | 349 | 0.901 | 0.809 | 0.771 | 0.729 |

**By Discipline**

| **Group** | **N** | **Total α** | **Supervision α** | **Autonomy α** | **Atmosphere α** |
| --- | --- | --- | --- | --- | --- |
| MBBS | 402 | 0.905 | 0.803 | 0.794 | 0.751 |
| BDS | 226 | 0.882 | 0.756 | 0.754 | 0.708 |
