# Supplementary material for: Contextual validation of HEMLEM tool used for measuring clinical micro-learning environments
Source: PLoS One. 2025 Dec 10;20(12):e0337641. doi: 10.1371/journal.pone.0337641 (PMC12694844; doi:10.1371/journal.pone.0337641)
Supplement: S7 Table — (DOCX) [file pone.0337641.s007.docx]

**SUPPLEMENTARY TABLE 7: RELIABILITY ANALYSIS**

**Table S7.1: Reliability Measures for HEMLEM 2.0 (N = 628)**

| **Scale/Subscale** | **Items** | **No. of Items** | **Cronbach's α** | **McDonald's ω** | **Composite Reliability (CR)** |
| --- | --- | --- | --- | --- | --- |
| **Total Scale** | Q1-Q12 | 12 | 0.897 | 0.929 | 0.901 |
| **Supervision** | Q1-Q4 | 4 | 0.787 | 0.815 | 0.792 |
| **Autonomy** | Q5-Q8 | 4 | 0.780 | 0.808 | 0.785 |
| **Atmosphere** | Q9-Q12 | 4 | 0.736 | 0.762 | 0.741 |

**Table S7.2: 95% Confidence Intervals for Reliability Measures**

| **Scale/Subscale** | **Cronbach's α [95% CI]** | **McDonald's ω [95% CI]** | **CR [95% CI]** |
| --- | --- | --- | --- |
| **Total Scale** | [0.883, 0.910] | [0.918, 0.939] | [0.888, 0.913] |
| **Supervision** | [0.758, 0.814] | [0.789, 0.839] | [0.764, 0.817] |
| **Autonomy** | [0.750, 0.807] | [0.781, 0.833] | [0.756, 0.811] |
| **Atmosphere** | [0.702, 0.767] | [0.731, 0.791] | [0.708, 0.771] |

**Table S7.3: Reliability by Demographic Groups**

**By Gender**

| **Scale/Subscale** | **Male (n=279)** |  |  | **Female (n=349)** |  |  |
| --- | --- | --- | --- | --- | --- | --- |
|  | Cronbach's α | McDonald's ω | CR | Cronbach's α | McDonald's ω | CR |
| **Total Scale** | 0.891 | 0.923 | 0.895 | 0.901 | 0.933 | 0.906 |
| **Supervision** | 0.758 | 0.788 | 0.764 | 0.809 | 0.835 | 0.813 |
| **Autonomy** | 0.791 | 0.818 | 0.795 | 0.771 | 0.799 | 0.776 |
| **Atmosphere** | 0.745 | 0.771 | 0.750 | 0.729 | 0.756 | 0.734 |

**By Academic Discipline**

| **Scale/Subscale** | **MBBS (n=402)** |  |  | **BDS (n=226)** |  |  |
| --- | --- | --- | --- | --- | --- | --- |
|  | Cronbach's α | McDonald's ω | CR | Cronbach's α | McDonald's ω | CR |
| **Total Scale** | 0.905 | 0.936 | 0.909 | 0.882 | 0.915 | 0.887 |
| **Supervision** | 0.803 | 0.830 | 0.808 | 0.756 | 0.785 | 0.761 |
| **Autonomy** | 0.794 | 0.821 | 0.798 | 0.754 | 0.783 | 0.759 |
| **Atmosphere** | 0.751 | 0.777 | 0.756 | 0.708 | 0.736 | 0.713 |
