# Supplementary material for: Contextual validation of HEMLEM tool used for measuring clinical micro-learning environments
Source: PLoS One. 2025 Dec 10;20(12):e0337641. doi: 10.1371/journal.pone.0337641 (PMC12694844; doi:10.1371/journal.pone.0337641)
Supplement: S8 File — (DOCX) [file pone.0337641.s008.docx]

**Supplementary File 8:**

**Participant Information Sheet**

**Part 1: Study Overview and Informed Consent**

**Study Title:** Contextual Validation of HEMLEM Tool Used for Measuring Clinical Micro-Learning Environments

**Introduction**

You are being invited to participate in a research study aimed at validating the HEMLEM tool, which measures clinical micro-learning environments in medical and dental education. This study will help us better understand and improve clinical placements for students.

**Purpose of the Study**

The purpose of this study is to evaluate the HEMLEM tool's ability to capture the experiences of students during their clinical placements and ensure its applicability in different educational contexts of Pakistan.

**Potential Risks and Benefits**

- **Risks:** Participation poses no physical or psychological risks.
- **Benefits:** While there are no direct personal benefits, your participation will contribute to provide a better understanding of clinical micro-learning education of Pakistan.

**Confidentiality and Data Privacy**

All information provided will be kept confidential and used solely for research purposes. Your responses will be anonymized, and no identifying details will be disclosed. Data will be securely stored and accessible only to the research team.

**Voluntary Participation and Withdrawal Rights**

Participation in this study is entirely voluntary. You may withdraw at any time without penalty or providing a reason.

**Contact Information**

If you have questions or concerns regarding this study, please contact:

Dr. Khizar Ansar Malik

Assistant Professor, Department of Medical Education, CMH Institute of Dentistry

Email: [khizarmalik1220@gmail.com](mailto:khizarmalik1220@gmail.com)

Phone number: 03005203056

**Consent Statement**

By signing below, you confirm that you have read and understood the study details provided above, and you consent to participate in this research.

- Yes, I consent to participate.
- No, I do not consent to participate.

Participant Signature: ________________________ Date: ______________________

**Part 2: Demographic Information**

Please provide the following information:

1. **Age: ______________**
2. **Gender:**
   - Male
   - Female
   - Other
3. **Field of Study:**
   - Medical
   - Dental
4. **Year of Study:**
   - 3rd Year
   - 4th Year
   - 5th Year
5. **Province of Study: ______________________**

**Part 3: HEMLEM Tool Questions**

Please rate the following statements based on your clinical placement experience using the 5-point Likert scale:

1 = Strongly Disagree | 2 = Disagree | 3 = Neutral | 4 = Agree | 5 = Strongly Agree

1. **This placement had a welcoming, friendly, and open atmosphere.**
2. **There was a culture where I felt free to ask questions or make comments on this placement.**
3. **Staff on this placement were enthusiastic about teaching.**
4. **My supervisor showed an interest in me.**
5. **My input was valued on this placement.**
6. **I was provided with regular, useful, and supportive feedback during this placement.**
7. **I had the opportunity to apply my previous knowledge in this placement.**
8. **My knowledge and skills were developed on this placement.**
9. **This placement helped me put theory into practice.**
10. **I was able to meet my learning objectives on this placement.**
11. **I had the opportunity to deal with the patient as a whole on this placement.**
12. **I was given tasks suitable for my stage of training on this placement.**

Thank you for your participation! Your input is valuable in improving clinical education.
