# Supplementary material for: Contextual validation of HEMLEM tool used for measuring clinical micro-learning environments
Source: PLoS One. 2025 Dec 10;20(12):e0337641. doi: 10.1371/journal.pone.0337641 (PMC12694844; doi:10.1371/journal.pone.0337641)
Supplement: Attachment — Submitted filename: point by point response.docx [file pone.0337641.s012.docx]

Response to editor Manuscript ID PONE-D-24-54864 entitled "Contextual validation of the HEMLEM tool used for measuring clinical micro-learning environments" submitted to PLOS ONE

Fatemeh Farshad

Guest Editor

PLOS ONE

Re: Manuscript ID PONE-D-24-54864

Many thanks for your careful consideration of our manuscript and thoughtful feedback. I have copied all the comments below and provided a detailed explanation of how we have addressed them. We have provided track changes and a clear version to reflect all the revisions. I am happy to make further revisions if you wish.

Best Regards

Ahsan Sethi (on behalf of the authors)

| **Sr#** | **Reviewer 1 Comments** | **Author response** |
| --- | --- | --- |
| 1 | This manuscript presents a validation study of the Healthcare Education Micro  Learning Environment Measure (HEMLEM 2.0) in Pakistani medical and dental education  contexts. While the study makes a potentially valuable contribution as a cross-cultural  validation effort, several important methodological concerns need to be addressed. | Thank you for your helpful and detailed feedback. We have added further details and made corrections to address any concerns. |
| 2 | Firstly, I couldn't find any information about language handling. The manuscript does not specify whether the instrument was translated from English or administered in English. If translation was used, the standard procedures of forward translation, back translation, expert committee review and cultural adaptation should be detailed. If the English version was used, justification and consideration of potential language barriers should be discussed. | Thank You for your comment. We have added the following details in the methodology section.  Subsection ‘Face Validity’:  “All students were provided with a participant information sheet that included a brief introduction to the research and its objectives, information about confidentiality and data privacy, a statement regarding voluntary participation and the right to withdraw, and a consent form. Students were also asked whether any items in the questionnaire were linguistically confusing or difficult to understand.”  AND in Subsection ‘Questionnaire’:  “The instrument was administered in English. Since English is the primary language of instruction at the participating institutions and no issues were reported during the validation phase, participants were proficient in English.” |
| 3 | “I noticed that the sampling approach is inadequately described. While the authors mention the use of a ‘convenience, non-probability sampling technique’ through WhatsApp groups and student IDs, important details are missing, such as: response rate calculations; justification for the final sample size of 628 participants; description of the total accessible population and the selection process for participating institutions.” | Thank you for this important observation. We have now expanded the **Methods** section to provide greater detail about our sampling approach. Specifically, we clarified that participants were recruited using a convenience, non-probability sampling method from **at least ten medical and dental schools representing all four provinces of Pakistan**. Outreach was conducted through institutional mailing lists, student WhatsApp groups, and student identification networks, with assistance from local academic coordinators.  While we aimed to achieve wide geographical coverage, the decentralized distribution of the survey through informal digital channels made it difficult to calculate an exact response rate or determine the total number of students contacted. We have clearly acknowledged this limitation in the revised manuscript and justified the sampling method based on accessibility and diversity of participating institutions. |
| 4 | From my perspective, the statistical analysis of this manuscript reveals both strengths and areas for improvement. The authors made appropriate use of confirmatory factor analysis and demonstrated strong internal 4consistency through reliability coefficients, w5ith an impressive omega coefficient of 0.927 and Cronbach's alpha of 0.895. Factor loadings are clearly presented, providing good transparency into the structure of the instrument. However, the model specification lacks sufficient detail to allow replication, and the authors have not explored alternative factor structures that might better explain the data. In addition, the lack of measurement invariance tests limits our understanding of how the instrument performs across different subgroups (e.g. dental vs. medical, female vs. male). | We sincerely thank the reviewer for highlighting critical areas for improvement. In response to the concerns raised, we have made the following significant revisions:   1. **Expanded CFA Model Specification:** We have now clearly articulated the structure of our three-factor CFA model in the Methods section, specifying the latent variables ("Supervision," "Autonomy," and "Atmosphere") and the observed items that load onto each. We have also clarified the estimation method, software used, and fit indices applied, which will enable full replication of our analysis. 2. **Comparison of Alternative Factor Structures:** To address the need for model comparison, we conducted additional CFA to compare three structures: a one-factor model, a three-factor model (our hypothesized structure), and a bifactor model. The results, show that the three-factor model demonstrated excellent fit (CFI = 0.951, RMSEA = 0.045), and the bifactor model only marginally improved fit. Given concerns about parsimony and interpretability, we retained the three-factor model as the most theoretically coherent and practically applicable. 3. **Measurement Invariance Testing Across Subgroups:** As recommended, we performed multi-group CFA to test measurement invariance across gender and academic discipline (medical vs. dental). Our analyses confirmed full configural, metric, and scalar invariance across both groups, indicating that the instrument operates equivalently across these demographic variables. These results are now presented in detail in the Results section, and discussed in the revised Discussion section with references to prior literature on invariance testing and cross-cultural validation. |
| 5 | For me, the limitations section is particularly concerning in its brevity and omission of important considerations. The use of convenience sampling introduces potential biases that should be acknowledged and discussed. The self-reported nature of the data and the online collection method using Google Forms present additional limitations that warrant discussion. The lack of test-retest reliability assessment leaves questions about the temporal stability of the instrument unanswered. Concerns about geographical generalisability and the lack of discipline specific analyses should also be addressed. | Thankyou for pointing this out. We have substantially revised the limitations section of the manuscript as follows:  “Despite its strengths, this study has several limitations that merit discussion. It employed a single-country design focused on Pakistan, which may restrict the generalisability of findings to other nations, even within similar clinical education frameworks. Future validation efforts should encompass a broader range of countries across the South-East Asian region and those with comparable clinical training systems.  While the study targeted both medical and dental students, it did not explore variations across specific specialties or departments. Such discipline-specific analyses could uncover unique factors influencing perceptions of the learning environment and should be considered in future research.  The use of convenience sampling via informal digital platforms (e.g., WhatsApp and email groups) limited the ability to define a clear sampling frame or calculate a response rate. Although participation was sought from all four provinces to enhance diversity, the non-random sampling approach may introduce selection bias and limit broader representativeness.  Furthermore, data collection through an online, self-administered Google Form may have excluded individuals with limited internet access or lower digital literacy, introducing additional sampling bias. The self-reported nature of the responses also makes the data vulnerable to social desirability and recall biases. Although the instrument demonstrated strong internal consistency, test-retest reliability was not assessed, leaving the temporal stability of responses unverified. Finally, while the geographical distribution of respondents was diverse, representation was uneven, and more balanced regional sampling would strengthen future studies” |
| 6 | Finally, please focus on the discussion section. It needs to be significantly expanded, particularly in terms of addressing limitations, practical implications and future research directions. | Thank you for your suggestion. We have revised the Discussion section accordingly. |
| 7 | In conclusion, I believe that this research demonstrates good methodological rigour and provides valuable insights for medical educators seeking to evaluate clinical microlearning environments, but taking into account the concerns presented above, I suggest the major revision. | Thank you for your encouraging and helpful feedback. Looking forward to its acceptance and publication |

| **Sr#** | **Reviewer 2 Comments** | **Author response** |
| --- | --- | --- |
| 1 | The article is well-written, and the rationale behind the selection and use of statistical analysis is well-understood, as well as the results discussed in a very good way.  The following are some points I would like to discuss with the team of authorship. | Thank you for your feedback. |
| 2 | Please add HEMLEM abbreviation in abstract. that will make it more clearer rather than to find it in the 3rd page. | Thank you for pointing this out. We have added this abbreviation in the abstract section |
| 3 | It appears that more descriptive analysis was conducted than what is mentioned according to Lines 175-186. Recommendation: Consider presenting these analyses either in a table format (xtable) or as an appendix to provide a clearer and more comprehensive view.. | Thank you for pointing out this important information. We have written the details of the descriptive statistics in the results section. We have also added table in the supplementary file 1. |
| 4 | Question: To what extent do you believe the online tool and Google Form can ensure equal opportunity for participation? Given concerns about internet access for students, how can we ensure there is no duplication (i.e., the same participant filling out the form multiple times) or students outside the criteria completing the form?  Recommendation: It would be beneficial to discuss these limitations in the article to address potential concerns regarding the validity of the data collected. | Thank you for your input. We have added the following in the methodology section:  “To minimize the risk of duplicate responses, the Google Form was set to restrict submissions to one response per Google account. Additionally, to ensure data completeness, all questionnaire items were marked as mandatory using the “required” option, which prevented respondents from submitting partially completed forms.”  However, access to the internet for all participants was a limitation in this study, and this issue has already been acknowledged in the Limitations section. |
| 5 | Correction: The document labeled as "APPENDIX 5" is referred to as "APPENDIX 4" within the document. Please correct this to maintain consistency. | Thank you for mentioning this. We have corrected the name accordingly |
