# Supplementary material for: Contextual validation of HEMLEM tool used for measuring clinical micro-learning environments
Source: PLoS One. 2025 Dec 10;20(12):e0337641. doi: 10.1371/journal.pone.0337641 (PMC12694844; doi:10.1371/journal.pone.0337641)
Supplement: Attachment — Submitted filename: Comment to Editor.docx [file pone.0337641.s010.docx]

**Comments to the Editor:**

This is a timely and important contribution to the field of global medical education, particularly in the under-researched area of micro-learning environments in LMICs. The authors demonstrate commendable rigor in their statistical approach and have made meaningful revisions to address earlier feedback. While the study has some limitations—particularly regarding sampling representativeness, test-retest reliability, and theoretical clarity of subscales—these are acknowledged and do not undermine the core contribution.

The manuscript is suitable for publication in PLOS ONE following minor revisions, which should focus on strengthening conceptual clarity, acknowledging methodological limitations, and offering more specific practical applications.
