# Supplementary material for: Contextual validation of HEMLEM tool used for measuring clinical micro-learning environments
Source: PLoS One. 2025 Dec 10;20(12):e0337641. doi: 10.1371/journal.pone.0337641 (PMC12694844; doi:10.1371/journal.pone.0337641)
Supplement: Attachment — Submitted filename: PLOS_ONE_Review_HEMLEM.docx [file pone.0337641.s011.docx]

Manuscript Title: Contextual validation of HEMLEM tool used for measuring clinical micro-learning environments

Comments to the Author:

This manuscript presents a well-executed, contextually grounded validation study of the HEMLEM 2.0 instrument for assessing clinical micro-learning environments in Pakistani medical and dental education. The study fills an important gap in the literature on culturally appropriate psychometric tools, particularly in low- and middle-income countries (LMICs). The authors have made substantial revisions that greatly improve the clarity, statistical transparency, and overall rigor of the study.
 **Major Strengths:**
Strong methodological rigor using Confirmatory Factor Analysis (CFA), bifactor modeling, and measurement invariance testing across gender and discipline.

- Inclusion of both Cronbach’s alpha and McDonald’s omega enhances the psychometric robustness of the findings.
- Cultural adaptation (e.g., changing “placement” to “department”) is thoughtful and contextually appropriate.
- Sampling from all four provinces and inclusion of both MBBS and BDS students increases generalisability within the Pakistani context.
- Measurement invariance testing supports the tool’s cross-group validity.

**Areas for Improvement:**
1. Test-Retest Reliability

- While the internal consistency is well established, the omission of test-retest reliability is a limitation. This should be more prominently acknowledged in both the discussion and limitations sections, along with recommendations for future research to assess the temporal stability of the tool.

2. Subscale Definitions

- The conceptual distinction between subscales—particularly “Autonomy” and “Teaching Quality”—is not sufficiently clear. Further explanation or reconsideration of the naming conventions would improve interpretability.

3. Linguistic and Cultural Sensitivity

- Although English is the medium of instruction, the paper would benefit from deeper reflection on potential language comprehension barriers. Consider elaborating on whether any support (e.g., bilingual review or pilot testing in native languages) was explored.

4. Practical Implications

- The discussion of how the validated HEMLEM 2.0 can be used for curriculum reform, rotation evaluation, and faculty development is promising but somewhat general. Including specific use-case examples (e.g., use in short rural clinical rotations or quality assurance dashboards) would enhance the applied relevance of the work.

5. Data Transparency

- While summary data are provided, uploading anonymized raw data and analysis code (e.g., R syntax) to a public repository such as OSF or Figshare would further strengthen transparency and reproducibility.

**Recommendation:** Minor Revision
