# Supplementary material for: Contextual validation of HEMLEM tool used for measuring clinical micro-learning environments
Source: PLoS One. 2025 Dec 10;20(12):e0337641. doi: 10.1371/journal.pone.0337641 (PMC12694844; doi:10.1371/journal.pone.0337641)
Supplement: Attachment — Submitted filename: Comments for authors.docx [file pone.0337641.s013.docx]

Dear Authors,

Thank you for your diligent work in revising the manuscript, "Contextual validation of HEMLEM tool used for measuring clinical micro-learning environments." Your comprehensive responses to the reviewers' comments have significantly enhanced the quality, clarity, and transparency of your work.

Here's a summary of how effectively you've addressed the feedback:

- **Clarity of Methodology and Applied Conclusions (Reviewer 3, Comment 1):** You have successfully clarified the distinct phases of your study in the "Materials & Methods" section, making the methodological approach much easier to follow. Furthermore, the expanded "Conclusion" section now provides more practical and functional implications of the HEMLEM 2.0 tool, which is a valuable addition.
- **Addressing Test-Retest Reliability (Reviewer 4, Comment 1):** You have appropriately acknowledged the omission of test-retest reliability as a limitation in the Discussion and have clearly recommended future longitudinal studies to assess the tool's temporal stability. This demonstrates a thorough understanding of the psychometric properties required for instrument validation.
- **Clarifying Subscale Definitions (Reviewer 4, Comment 2):** Your detailed explanation differentiating "Autonomy" and "Teaching Quality" in the "Preliminary Psychometric Testing and Exploratory Analysis" subsection is very helpful. This clarification significantly improves the interpretability of these conceptually distinct subscales.
- **Enhancing Linguistic and Cultural Sensitivity (Reviewer 4, Comment 3):** The added description of cognitive interviews, where you explicitly explored potential language comprehension barriers with students, addresses this point effectively. Your commitment to ensuring linguistic accessibility and cultural relevance through multiple rounds of student feedback is commendable.
- **Providing Specific Practical Implications (Reviewer 4, Comment 4):** You have successfully incorporated more concrete use-case examples for the HEMLEM 2.0 tool in the "Discussion" section. The suggestions for integrating it into digital dashboards, using it in rural clinical placements, and informing faculty development initiatives make the applied relevance of your work much clearer.
- **Improving Data Transparency (Reviewer 4, Comment 5):** Your decision to make the anonymized dataset publicly available on Zenodo, along with the direct link in the "Data Availability Statement," is an excellent step. This significantly boosts the transparency and reproducibility of your research, aligning with best practices in scientific publishing.

Overall, your revisions have substantially strengthened the manuscript. The added details, clarifications, and increased transparency reflect a thorough and thoughtful engagement with the reviewers' feedback.
