# Supplementary material for: Contextual validation of HEMLEM tool used for measuring clinical micro-learning environments
Source: PLoS One. 2025 Dec 10;20(12):e0337641. doi: 10.1371/journal.pone.0337641 (PMC12694844; doi:10.1371/journal.pone.0337641)
Supplement: Attachment — Submitted filename: Comments for editor.docx [file pone.0337641.s014.docx]

I have reviewed the revised manuscript, "Contextual validation of HEMLEM tool used for measuring clinical micro-learning environments" (PONE-D-24-54864R2), and the authors' detailed response to reviewers. Overall, the authors have made substantial efforts to address the feedback provided, significantly improving the manuscript's clarity, rigor, and transparency.

Here's a breakdown of how the authors have addressed each reviewer's comment:

**Reviewer 3 Comments:**

1. **"Explain more clearly about methodology, especially phases of the study. Also, please extend conclusions of the study more functional and applied."**

- **Authors' Response:** "Thank you for your suggestions. We have now made revision to reflect clarity in the various phases of the study. We have also extended the conclusions section to make it more functional and applied."
- **Assessment:** The authors have indeed revised the "Materials & Methods" section (lines 120-165 for Phase 1 and 166-242 for Phase 2) to clearly delineate the two distinct phases of the study: "Phase 1: Methodological Part" (cultural adaptation, content, and face validation) and "Phase 2: Cross-Sectional Phase: Construct Validation & Reliability Testing." The "Conclusion" section (lines 490-500) has also been expanded to include more functional and applied implications, such as using HEMLEM 2.0 for "academic audits, quality assurance, and faculty development," and its integration into "routine evaluations and digital dashboards." **This comment has been adequately addressed.**

**Reviewer 4 Comments:**

1. **"Test-Retest Reliability: While the internal consistency is well established, the omission of test-retest reliability is a limitation. This should be more prominently acknowledged in both the discussion and limitations sections, along with recommendations for future research to assess the temporal stability of the tool."**

- **Authors' Response:** "Thank you for pointing this out. We have revised the discussion section by adding these lines; 'Furthermore, although the study confirmed strong internal consistency and robust construct validity, it did not include an assessment of test-retest reliability. This omission means the temporal stability of the instrument remains unverified. Without repeated measurements over time, it is unclear whether HEMLEM 2.0 consistently captures student perceptions across different points in the academic year or during varied clinical exposures. Future studies should adopt longitudinal designs to assess reliability over time, especially if the tool is to be used for ongoing monitoring or benchmarking.'"
- **Assessment:** The authors have incorporated a clear acknowledgment of the lack of test-retest reliability as a limitation in the Discussion section (lines 467-473) and have explicitly recommended future longitudinal studies to address this (lines 471-473, and reiterated in future research recommendations, lines 480-481). **This comment has been adequately addressed.**

2. **"Subscale Definitions: The conceptual distinction between subscales—particularly 'Autonomy' and 'Teaching Quality'—is not sufficiently clear. Further explanation or reconsideration of the naming conventions would improve interpretability."**

- **Authors' Response:** "Thank you for this suggestion, we have added details in the methods section: 'Although the final factor structure supported a three-domain model—Supervision, Autonomy, and Atmosphere—additional clarification was necessary to distinguish between the constructs of Autonomy and Teaching Quality, which may appear conceptually overlapping. In this context, Autonomy reflects the extent to which students perceived opportunities to engage in self-directed clinical tasks, apply their existing knowledge, and participate in patient care with increasing independence. On the other hand, Teaching Quality captures the perceived effectiveness, clarity, and engagement of clinical instruction provided by supervisors. These subscales were retained based on both theoretical rationale and item alignment, though future revisions may consider refinement of labels to enhance interpretability.'"
- **Assessment:** The authors have provided a more detailed explanation of the conceptual distinction between "Autonomy" and "Teaching Quality" in the "Preliminary Psychometric Testing and Exploratory Analysis" subsection (lines 298-307). This clarification enhances the interpretability of these subscales. **This comment has been adequately addressed.**

3. **"Linguistic and Cultural Sensitivity: Although English is the medium of instruction, the paper would benefit from deeper reflection on potential language comprehension barriers. Consider elaborating on whether any support (e.g., bilingual review or pilot testing in native languages) was explored."**

- **Authors' Response:** "Thank you for the suggestion, We have clarified this in the methodology section: 'Additionally, cognitive interviews were conducted with 10 students using a structured debriefing format to assess the clarity and comprehensibility of the questionnaire. Each participant received a detailed information sheet outlining the study's objectives, confidentiality measures, voluntary participation, and consent procedures. Students were specifically asked to identify any linguistically confusing or unclear items. Although English is the medium of instruction at all participating institutions, potential language barriers were directly explored during this phase. No significant comprehension issues were reported. While a bilingual version was not piloted, the tool underwent multiple rounds of student feedback to ensure linguistic accessibility and cultural relevance for the target population.'"
- **Assessment:** The authors have added a detailed explanation of the cognitive interviews conducted with students to assess clarity and comprehensibility, explicitly stating that potential language barriers were explored and no significant issues were reported (lines 157-165). They also clarify that a bilingual version was not piloted but extensive student feedback was incorporated. **This comment has been adequately addressed.**

4. **"Practical Implications: The discussion of how the validated HEMLEM 2.0 can be used for curriculum reform, rotation evaluation, and faculty development is promising but somewhat general. Including specific use-case examples (e.g., use in short rural clinical rotations or quality assurance dashboards) would enhance the applied relevance of the work."**

- **Authors' Response:** "Thank you for your suggestion. We have revised the discussion section accordingly."
- **Assessment:** The "Discussion" section now includes more specific examples of how HEMLEM 2.0 can be applied, such as integration into "digital dashboards to provide department-level feedback," its use as a "diagnostic tool to assess whether essential educational elements are preserved in decentralized environments" like short-term rural clinical placements, and informing "targeted faculty development initiatives" (lines 446-455). **This comment has been adequately addressed.**

5. **"Data Transparency: While summary data are provided, uploading anonymized raw data and analysis code (e.g., R syntax) to a public repository such as OSF or Figshare would further strengthen transparency and reproducibility."**

- **Authors' Response:** "Thank You for your suggestion. We have included this in our manuscript: 'Data Availability Statement: The anonymized dataset used in this study are available at <https://zenodo.org/records/15616880>. The files are openly accessible and support full reproducibility of our findings.'"
- **Assessment:** The authors have commendably made their anonymized dataset publicly available on Zenodo, providing a direct link in the "Data Availability Statement" (lines 509-511). This significantly enhances the transparency and reproducibility of their findings. **This comment has been fully addressed.**

In conclusion, the authors have diligently responded to all reviewer comments, making appropriate and substantial revisions to the manuscript. The updated version is significantly improved and is now suitable for publication.
