# Supplementary material for: Contextual validation of HEMLEM tool used for measuring clinical micro-learning environments
Source: PLoS One. 2025 Dec 10;20(12):e0337641. doi: 10.1371/journal.pone.0337641 (PMC12694844; doi:10.1371/journal.pone.0337641)
Supplement: Attachment — Submitted filename: Response to Reviewers - Fourth Round 250925.docx [file pone.0337641.s017.docx]

Response to editor Manuscript ID PONE-D-24-54864R3 - EMID:2eee3f8f87e5fc7a entitled "Contextual validation of the HEMLEM tool used for measuring clinical micro-learning environments" submitted to PLOS ONE

Fatemeh Farshad

Guest Editor

PLOS ONE

Re: Manuscript ID PONE-D-24-54864R3 - EMID:2eee3f8f87e5fc7a

Many thanks for your careful consideration of our manuscript and thoughtful feedback. I have copied all the comments below from reviewer# 5 and provided a detailed explanation of how we have addressed them. We have provided a copy with highlighted changes and a clear version to reflect all the revisions. I am happy to make further revisions if you wish.

Best Regards

Ahsan Sethi (on behalf of the authors)

| **Reviewer 5 Comments** | **Author response** |
| --- | --- |
| This manuscript describes the cultural adaptation and psychometric validation of the HEMLEM tool for use in Pakistani medical and dental schools. The study addresses an important gap in the literature concerning the assessment of clinical micro-learning environments in a new cultural context. The work is generally well-structured, and the authors have undertaken a rigorous process for content validation. The use of advanced statistical techniques like Multi-Group Confirmatory Factor Analysis (MGCFA) for measurement invariance is a significant strength. However, several critical methodological and statistical concerns, particularly regarding the conflation of Exploratory Factor Analysis (EFA) and Confirmatory Factor Analysis (CFA), undermine the validity of the reported findings. These issues require substantial revision. | Thank you. We have addressed all your comments in the revised manuscript. |
| Major Conceptual and Statistical Flaw: Conflation of EFA and CFA  This is the most critical issue in the manuscript. The authors state that their primary analysis was CFA to test an a priori three-factor model. However, the methodology and results sections are heavily, and confusingly, reliant on EFA techniques.  Page 21, Line 293-303; Page 22, Table 3: The presentation of a "Rotated Component Matrix" using Principal Component Analysis (PCA) with Varimax rotation is a purely exploratory technique. PCA is a data reduction method, not a latent variable modeling technique like EFA or CFA. Presenting this after claiming to have run a CFA creates a contradictory narrative.  Justification in Response to Reviewers (Page 2-3): The authors' response to Reviewer 5, while defending the use of CFA, acknowledges that Table 3 displays "preliminary loadings for reader transparency." These exploratory values should not be the basis for assigning items to domains (as done on Lines 298-300: "based on the factor loading values, items 1 till 6 were assigned to domain 1..."). In a true CFA, the factor structure (which items load on which factor) is pre-specified based on theory or prior EFA, not determined from the current dataset.  Recommendation: The authors must clarify their analytical strategy. If an EFA/PCA was indeed conducted on the Pakistani dataset to explore the factor structure, this must be explicitly stated in the methods before the CFA is introduced. The results of the EFA should be presented first, followed by the CFA which tests the structure suggested by the EFA (or the original theoretical structure). The current presentation suggests a circular analysis where the same data is used to explore and then confirm the structure, which is methodologically unsound. If the CFA was truly theory-driven and confirmatory, the EFA/PCA results (Table 3) should be removed entirely, as they are redundant and misleading. | We thank the reviewer for this critical and helpful observation. Based on this feedback, we have entirely removed Table 3 and all references to PCA or Exploratory analysis from both the Methods and Results sections. The current manuscript follows a strictly confirmatory approach, with a pre-specified three-factor model derived from the original theoretical structure of the HEMLEM instrument. All item-to-factor assignments were made a priori, and no data-driven reallocation was performed. The CFA was conducted solely to test the applicability of this established structure in a new cultural context. The revised Statistical Analyses and Results sections now reflect this strategy with added clarity, ensuring there is no conflation of exploratory and confirmatory methods. |
| **2**. Sample Size Justification  Page 17, Lines 201-202: The justification ("10 participants per item") is outdated and not a robust methodological standard. While the final sample size of N=628 is excellent for a 12-item scale and provides high statistical power, the justification should be updated with contemporary references that consider desired factor loading magnitudes, number of factors, and anticipated communalities (e.g., Kyriazos, 2018; Wolf et al., 2013).  Page 25, Lines 390-399: The sample size discussion in the manuscript is better, citing Comrey & Lee and others. This stronger justification should be moved to the Methods section. | We appreciate this insightful comment. The outdated "10 participants per item" rule has been removed from the Methods section. Instead, we have incorporated a more comprehensive justification that reflects current psychometric standards, including citations to Wolf et al. (2013) and Kyriazos (2023). The revised paragraph has been relocated to the ‘Sample Size’ subsection in the Methods section, as recommended. The paragraph in the ‘Discussion’ section has also been revised accordingly |
| 3. Measurement Invariance Reporting  Page 24, Lines 334-344: The MGCFA is a strength. However, the results are not sufficiently detailed. The authors must report the fit indices (χ², df, CFI, TLI, RMSEA, SRMR) for the configural, metric, and scalar models for both gender and discipline in the main text or a table, not just refer to supplemental materials. The critical values for evaluating invariance (ΔCFI < 0.01, ΔRMSEA < 0.015) should be stated in the Methods section. | We thank the reviewer for this valuable observation.  We have added full model fit indices (χ², df, CFI, TLI, RMSEA, SRMR) for configural, metric, and scalar models across both gender and academic discipline groups.  These results are now presented within the main text and supported by two newly created tables (Tables 3 and 4) that clearly display the MGCFA outputs. |
| 4. Reliability Reporting  Page 24, Line 346-347: Reporting both Cronbach's alpha and McDonald's omega is a best practice. However, the value of "composite reliability" (0.766) is mentioned without a clear definition. It should be specified that this is likely the Maximal Reliability for the general factor (H) if referring to the bifactor model, or the construct reliability for the specific factors. The calculation formula (e.g., Raykov's rho) should be referenced. | We thank the reviewer for this important observation regarding the reporting of composite reliability.  We have added a clear description of how composite reliability was calculated in the methods section.  We have corrected an error in our original calculation and updated the text in results section under the ‘Reliability’ subsection  We have also added a comprehensive table in the supplementary file (Table S7) showing all three reliability measures (Cronbach's α, McDonald's ω, and CR).  We note that the originally reported CR value of 0.766 was incorrectly calculated. The correct value based on CFA factor loadings is 0.901, which aligns better with the other reliability measures and indicates excellent construct reliability. |
| 5. Terminology and Clarity  Page 22, Lines 304-312: The distinction between "Autonomy" and "Teaching Quality" is helpful. However, the manuscript later reverts to the original three-factor model labels ("Supervision, Autonomy, Atmosphere"). There is an inconsistency between the two-domain structure mentioned in the EFA section (Staff Attitudes/Teaching Quality) and the three-domain CFA structure. This discrepancy must be explicitly addressed and resolved. The labels used in the final model must be consistent throughout the paper. | We appreciate the reviewer’s observation regarding inconsistent domain labels. In response, we have removed the exploratory terminology ("Teaching Quality", "Staff Attitudes") that appeared in earlier drafts and now consistently refer to the original HEMLEM domains: Supervision, Autonomy, and Atmosphere. |
| 6. Data Analysis Description  Page 18, Lines 233-242; Page 24, Lines 334-344: The description of MGCFA is good. However, the software mention on Page 24, Line 245 (R with Lavaan) contradicts the earlier statement on Page 18, Line 213 (IBM SPSS AMOS). This must be corrected for consistency.  Page 18, Line 214: The assessment of normality via skewness and kurtosis is appropriate. It would be beneficial to state the specific cut-off values used (e.g., \|skewness\| < 2, \|kurtosis\| < 7). | We thank the reviewer for identifying the inconsistency in the software description. We have revised the Methods section to correctly state that CFA and MGCFA were conducted using R with the Lavaan package, while descriptive statistics were performed using SPSS.  We have also clarified the criteria for assessing normality by specifying that acceptable skewness and kurtosis values were set at < 2 and < 7, respectively, based on standard guidelines. |
| 7. Limitations  Page 27-28, Lines 480-497: The limitations section is comprehensive and well-written. It appropriately acknowledges the sampling method, lack of test-retest reliability, and potential for bias.  Summary of Required Revisions Clarify the Factor Analysis Sequence: Decide and clearly report whether an EFA was conducted. If yes, present EFA results first, then use them to inform the CFA model. If the study was purely confirmatory, remove the EFA/PCA results (Table 3) and all references to using the current data to assign items to factors.  Resolve Terminology Inconsistencies: Ensure the names of the factors and subscales are consistent between the EFA (if kept) and CFA sections and throughout the manuscript.  Provide Detailed MGCFA Results: Report full fit indices for all invariance models in the main text.  Improve Sample Size Justification: Replace the "10 per item" rule with a more contemporary justification in the Methods section.  Correct Software Inconsistencies: Ensure the software used for each analysis is correctly and consistently reported.  Minor Proofreading: Address minor grammatical errors and ensure consistency in spelling (e.g., "generalizability" vs. "generalisability").  Conclusion The authors have undertaken a valuable study with considerable methodological strengths, including a strong cultural adaptation process, a large sample, and the application of advanced statistical techniques like MGCFA. However, the fundamental confusion between exploratory and confirmatory analysis paradigms is a major flaw that affects the interpretation and validity of the core findings. Addressing this central issue is paramount. Once these major revisions are complete, this manuscript has the potential to be a significant contribution to the field of health professions education. | We thank the reviewer for their thorough and constructive feedback. We have carefully addressed each of the concerns noted in the summary |
