# Supplementary material for: Contextual validation of HEMLEM tool used for measuring clinical micro-learning environments
Source: PLoS One. 2025 Dec 10;20(12):e0337641. doi: 10.1371/journal.pone.0337641 (PMC12694844; doi:10.1371/journal.pone.0337641)
Supplement: Attachment — Submitted filename: Response to Reviewers - Fifth Round 061125.docx [file pone.0337641.s018.docx]

Response to editor Manuscript ID PONE-D-24-54864R4 entitled "Contextual validation of the HEMLEM tool used for measuring clinical micro-learning environments" submitted to PLOS ONE

Fatemeh Farshad

Guest Editor

PLOS ONE

Re: Manuscript ID PONE-D-24-54864R4

Many thanks for your careful consideration of our manuscript and thoughtful feedback. I have copied all the comments below from reviewer# 5 and provided a detailed explanation of how we have addressed them. We have provided a copy with highlighted changes and a clear version to reflect all the revisions. I am happy to make further revisions if you wish.

Best Regards

Ahsan Sethi (on behalf of the authors)

| **Editor Comments** | **Author response** |
| --- | --- |
| Due to the reviewer's comment, It is recommended to revise by an English professional editor. | Thank you for your message. Unfortunately, we do not have any funds allocated for this project or for professional English editing services. Our team has extensive publication experience and a strong command of the English language.  After five rounds of revisions and feedback from six reviewers, we have carefully re-examined the manuscript and believe that the English is appropriate for publication. Unless there are specific areas that require further language improvement, we would kindly request that the paper be considered for acceptance at this stage. |
| **Reviewer 6 Comments** | **Author response** |
| It is a good topic. After 4 rounds and review, it has been better.  It is recommended to revise by an English professional editor. (Minor revision). | Thank you. Unfortunately, we do not have any funds allocated for this project or for professional English editing services. Our team has extensive publication experience and a strong command of the English language.  After five rounds of revisions and feedback from six reviewers, we have carefully re-examined the manuscript and believe that the English is appropriate for publication. Unless there are specific areas that require further language improvement, we would kindly request that the paper be considered for acceptance at this stage. |
